# PD-L1 expression in pancreatic ductal adenocarcinoma is a poor prognostic factor in patients with high CD8^+^ tumor-infiltrating lymphocytes: highly sensitive detection using phosphor-integrated dot staining

**DOI:** 10.1007/s10147-017-1112-3

**Published:** 2017-03-18

**Authors:** So Yamaki, Hiroaki Yanagimoto, Koji Tsuta, Hironori Ryota, Masanori Kon

**Affiliations:** 1 Department of Surgery, Kansai Medical University Hirakata Hospital, 2-3-1 Shinmachi, Hirakata-Shi, Osaka 573-1191 Japan; 2 Department of Pathology and Laboratory Medicine, Kansai Medical University, Hirakata, Osaka Japan

**Keywords:** Pancreatic adenocarcinoma, PD-L1, Immunohistochemistry, Phosphor-integrated dots

## Abstract

**Background:**

Pancreatic ductal adenocarcinoma (PDAC) has an extremely poor prognosis. For the development of more effective immunotherapies, it is first necessary to elucidate the immunological escape mechanisms. In this study, we applied our recently developed highly sensitive immunostaining method employing fluorescent phosphor-integrated dot (PID) nanoparticles to evaluate the prevalence of programmed death ligand 1 (PD-L1) in patients with PDAC.

**Methods:**

This study included 42 patients with PDAC who underwent pancreatectomy. We evaluated PD-L1 expression in these patients using PID staining and correlated PD-L1 expression level with each patient’s clinico-pathological features.

**Results:**

PD-L1 expression was detected in 61.9% (26/42) of the patients with PDAC by PID staining. There was a significant difference in overall survival between PD-L1-positive and PD-L1-negative patients [hazard ratio (HR) 2.07, 95% confidence interval (CI) 1.00–4.54; *P* = 0.049]. Among CD8^+^-tumor-infiltrating lymphocyte-positive cases, the overall survival of PD-L1-positive patients was significantly poorer than that of PD-L1-negative patients (HR 3.84, 95% CI 1.59–10.35; *P* = 0.003). Univariate and multivariate analyses indicated that PD-L1 expression was an independent predictive poor prognostic factor in patients with PDAC.

**Conclusions:**

PD-L1 expression appears to be an important prognostic factor in patients with PDAC who underwent surgical resection.

## Introduction

Pancreatic ductal adenocarcinoma (PDAC) is a lethal disease with a poor prognosis; it is the fourth leading cause of cancer-related deaths in Japan and the fourth most common malignancy in the USA [1, 2]. At the time of PDAC diagnosis, less than 20% of the tumors are resectable, and the actual 5-year survival rate is reported to range from 15 to 25% [3]. Current clinical treatments for PDAC have limited efficacy; thus, improved treatment strategies are required to prolong patient survival.

Expression of programmed death-1 (PD-1) is significantly upregulated on activated cancer-specific T cells. The PD-1 receptor attaches to its ligand PD-L1, which is expressed by tumor cells and infiltrating immune cells. The interaction of PD-1 and PD-L1 inhibits T-cell activation and promotes tumor immune escape [4–7]. The escape mechanism acquired by tumor cells to avoid immune recognition and destruction is a major contributor to the limitations of the therapeutic efficacy. However, recently developed therapeutic antibodies against PD1/PD-L1 show promising clinical results for several tumors, such as melanoma, renal cancer, and non-small cell lung cancer [8].

Although the efficacy of PD-1/PD-L1 antibody therapy should be correlated with PD-L1 protein expression in tumor cells, approximately 10–40% of PD-L1 immuno-negative cases also respond to anti-PD-1/PD-L1 therapy [9–11]. This contradiction may be caused by the performance of the PD-L1 immunostaining assay which is based on the color intensity visualized using the chromogen dye diaminobenzidine (DAB). We recently developed an immunohistochemistry method using fluorescence-emitting phosphor-integrated dot (PID) nanoparticles as a fluorescent dye. PID shows a higher luminance and dynamic range than those of conventional fluorescent dyes and DAB [12]. Specifically, the fluorescence intensity of the PID particles was found to be approximately 100-fold higher than that of a conventional fluorescent dye. Also, the ratio of particle to antibody binding is 1:1. Thus, this technique is highly sensitive as well as quantitative as compared to the conventional DAB-based method.

The aim of the study reported here was to evaluate the expression of PD-L1 in patients with PDAC by immunostaining using PID technology and to compare the results with those obtained after conventional DAB staining.

## Materials and methods

### Patients and samples

This study included 42 patients with PDAC, of whom 31 underwent pancreaticoduodenectomy and 11 underwent distal pancreatectomy at the Department of Surgery at Kansai Medical University Hospital (Osaka, Japan) between May 2001 and December 2007. All patients had histologically confirmed PDAC. The tumors were classified according to the TNM classification [13]. The clinical parameters of all patients were collected from a prospectively maintained institutional PDAC database.

Surgically resected specimens were fixed in formalin and embedded in paraffin; and serial sections cut from the embedded specimens were stained with hematoxylin and eosin for histological evaluation. The most representative tumor areas were sampled for the tissue microarray using 2-mm-diameter samples (Azumaya, Tokyo, Japan).

This study was conducted in accordance with the Declaration of Helsinki, and the study protocol was approved by the institutional review board of our hospital (Protocol no. H151043 and 27-14).

### Immunohistochemistry

Four-micrometer-thick sections were deparaffinized using routine procedures. Endogenous peroxidase activity in the deparaffinized sections was blocked by treating the sections with 3% hydrogen peroxide treatment for 15 min, followed by washing in deionized water for 2–3 min. The sections were subsequently boiled in 10 mM sodium citrate buffer for 10 min at 121 °C, then allowed to cool at room temperature for 40 min, followed by rinsing with deionized water and washing with phosphate-buffered saline for 5 min.

### Measurement of the fluorescence properties of PID

**Fig. 1:**
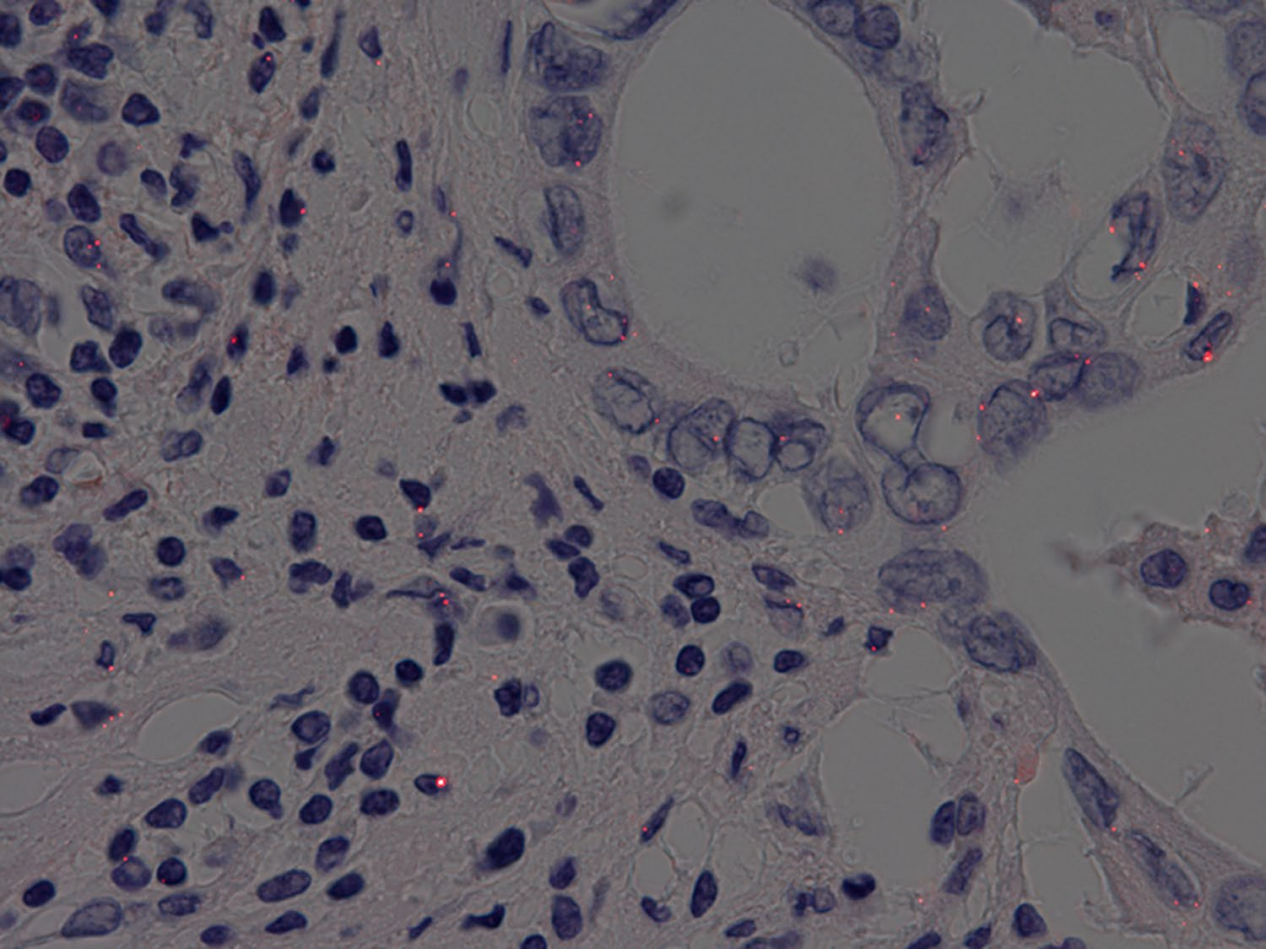
Immunohistochemistry of pancreatic ductal adenocarcinoma tissue using phosphor-integrated dot (PID) staining. *Red spots* on tumor cells indicate PID particles

The sections were incubated with the primary antibody toward PD-L1 (E1L3N, 1:1000, Cell Signaling Technology, Danvers, MA). Sections were incubated with 2 μg/mL biotinylated anti-rabbit antibody (LO-RG-1) for 30 min and then with PID-conjugated streptavidin (0.06 nM) for 2 h, both at room temperature. The sections were then irradiated at 580 nm, and the fluorescence intensities were measured using a BX53 fluorescence microscope (Olympus, Tokyo, Japan); images were acquired with a DP73 CCD camera (Olympus) (Fig. 1). The number of PID particles per cell was measured with an automated PID Analyzer (Konica Minolta, Tokyo, Japan). The number of PD-L1 particles was evaluated only on the tumor cells. Five fields at 400× magnification were selected randomly, and the number of PD-L1 particles on each tumor cell was counted and the average number of particles per cell then calculated for each field. The highest value among the five fields was determined to be the PID staining value. The negative control was prepared with PID staining but without the primary antibody.

### Measurement of DAB intensity

The sections were incubated with the primary antibody toward PD-L1 (E1L3N, 1:200, Cell Signaling Technology) diluted in antibody diluent (Signal Stain Antibody Diluent #8112; Cell Signaling Technology). The sections were treated with peroxidase-labeled secondary antibody (EnVision/HRP system; DAKO, Carpinteria, CA) after linker reagent treatment for 15 min. The sections were then rinsed in the buffer and immersed in DAB to observe color development.

For the DAB-naked eye evaluation, three observers (S.Y., H.R., K.T.) assessed the immunostaining results in a blinded manner without knowledge of the clinical or histopathological diagnoses. Intensity was graded on a 3-tier scale (1+, negative to weak; 2+, moderate; 3+, strong). The percentage of staining was recorded, and a semi-quantitative (H-score) approach [14] was used for analysis. PD-L1 expression scores were calculated (from 0 to 300) by multiplying the percentage of the stained tumor area by the staining intensity score.

### Double staining of PD-L1 using the PID method and of CD8^+^ lymphocytes using the DAB method

**Fig. 2:**
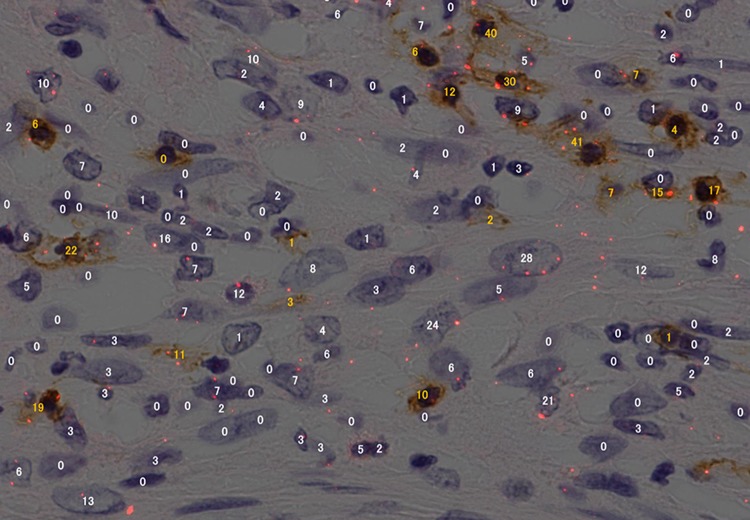
Immunohistochemical double staining for programmed death ligand 1 (PD-L1) on tumor cells and CD8^+^ lymphocytes. PID staining was used for PD-L1 detection, and diaminobenzidine (DAB) staining was used for CD8+ lymphocytes. The number of PID particles measured by the automated PID analyzer is indicated for each tumor cell (*white numbers*) and lymphocyte (*yellow numbers*)

Double staining, i.e., DAB staining for CD8^+^ lymphocytes and PD-L1 staining with the PID method, was performed to count the tumor infiltrating lymphocytes (TILs) (primary antibody for PD-L1: E1L3N, 1:100; primary antibody for CD8: C8/114B, 1:250; DAKO; secondary antibody: EnVision/HRP system) (Fig. 2).

The correlations between PD-L1 expression, overall survival, and clinico-pathological data were evaluated.

### Statistical analysis

Statistical analysis was performed using JMP® 10 software (SAS institute Inc., Cary, NC). Student’s *t* test was used to analyze continuous variables, and the χ^2^ test was used to analyze categorical variables. Cumulative survival rates were calculated by the Kaplan–Meier method. Significant differences in survival status were evaluated using the log-rank test. The Cox proportional hazards model was used in the multivariate analysis, and values are expressed using the hazard ratio (HR) with a 95% confidence interval (CI). *P* < 0.05 was considered to be statistically significant.

## Results

### Patient characteristics

**Fig. 3:**
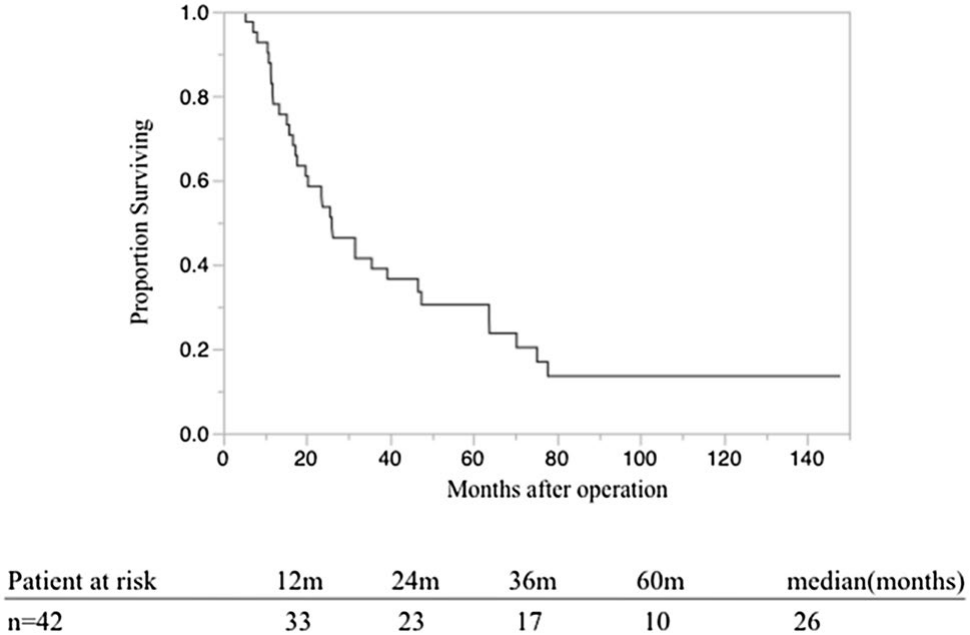
Overall survival of 42 patients with pancreatic ductal adenocarcinoma (PDAC)

The patient background and clinico-pathological parameters are shown in Table 1. Of the 42 patients in the study, 16 were women and 26 were men, with a median age at the time of diagnosis of 65s (range 50–83) years. In terms of TMN stage at diagnosis, three, six, 33, and zero patients were diagnosed at the T1, T2, T3, and T4 stages, respectively. Lymph node metastases were detected in 26 of the patients (61.9%). Seven patients were identified as category M1 because of metastasis of the number 16 lymph node without other organ metastasis. In terms of the pathological stage (pStage), as defined in the Union for International Cancer Control classification, three, four, nine, 19, and seven cases were at pStage Ia, Ib, IIa, IIb, and IV, respectively. The median survival time (MST) of the 42 patients was 26 months (Fig. 3).

**Table 1:** Patient characteristics and clinico-pathological data

Patient characteristics	Values (*n* = 42 patients)
Age (years)	65 (50–83)
Male/female	26/16
Tumor location (Ph/Pbt)	31/11
R0/R1^a^	27/15
Neo-adjuvant therapy (+/−)	8/34
Adjuvant therapy (+/−)	23/19
Pre-operative tumor marker CA19-9	134.2 (1.6–8116)
T stage (½/3)^b^	3/6/33
N stage (0/1)^b^	16/26
M stage (0/1)^c^	35/7
Tumor diameter (mm)	30 (16–75)

The data in table are expressed as the median with the range in parenthesis or as the number of patients

*Ph* Pancreatic head, *Pbt* pancreatic body or tail

^a^R0 corresponds to curative resection or complete remission; R1 corresponds to microscopic residual tumor

^b^T and N stage were based on the TNM classification of malignant tumors, sixth edition

^c^All M1 cases had No.16 lymph node metastasis without other organ metastasis

Of the 42 patients, 27 (64.3%) underwent pathological curative resection, eight (19.0%) received neo-adjuvant chemo-radiation therapy, and 23 (54.8%) received adjuvant chemotherapy.

### PD-L1 expression in the 42 PDAC patients by DAB and PID staining

To judge the rate of positive PID staining, we first established the threshold of the PID staining value. The average of the highest value of the negative control of PID staining was 3.01; therefore, the threshold value for judging positive PID staining was set to 3.0. Using this threshold, we detected PD-L1 expression in 26 of the 42 patients (61.9%).

By contrast, PD-L1 expression measured by the DAB-naked eye evaluation was detected in only six of the 42 patients (14.3%). This expression rate was measured to be between the PID positive staining value derived from the threshold set at 4.0 (28.6%, 12/42 patients) and 5.0 (11.9%, 5/42 patients).

### Relationship between PD-L1 expression and clinico-pathological features

The correlations between pathological features and PD-L1 expression are shown in Table 2.

**Table 2:** Relationships between programmed death ligand 1 expression and pathological features

Parameter^a^	PD-L1 expression (+) (*n* = 26)	PD-L1 expression (−) (*n* = 16)	*P*
Age (years)	64 (51–82)	66 (50–78)	0.385
Male/female	20/6	6/10	0.021
Tumor location (Ph/Pbt)	19/7	12/4	0.891
R0/R1	15/11	12/4	0.256
Neo-adjuvant therapy (+/−)	5/21	3/13	0.969
Adjuvant therapy (+/−)	15/11	8/8	0.627
Pre-operative tumor marker CA19-9	143.0 (1.6–8116)	113.0 (20.3–1712)	0.421
T stage (1, 2/3)	5/21	4/12	0.711
N stage (0/1)	10/16	6/10	0.950
M stage (0/1)	21/5	14/2	0.570
Tumor diameter (mm)	29.5 (18–75)	32.5 (16–45)	0.583

The data in table are expressed as the median with the range in parenthesis or as the number of patients

*PD-L1* Programmed death ligand 1

^a^See footnotes to Table 1 for explanations of staging

The ratio of males was significantly higher in the PD-L1-positive group (male/female 20/6) than in the PD-L1-negative group (male/female: 6/10 (*P* = 0.021). There was no significant correlation between PD-L1 expression and tumor size, lymph node metastasis [including distant lymph node metastasis (M1)], pre-operative tumor marker CA19-9 level, and R0/R1 (microscopic residual tumor) status. CD8^+^ TIL counts were not significantly correlated to PD-L1 expression.

### Survival analysis

**Fig. 4:**
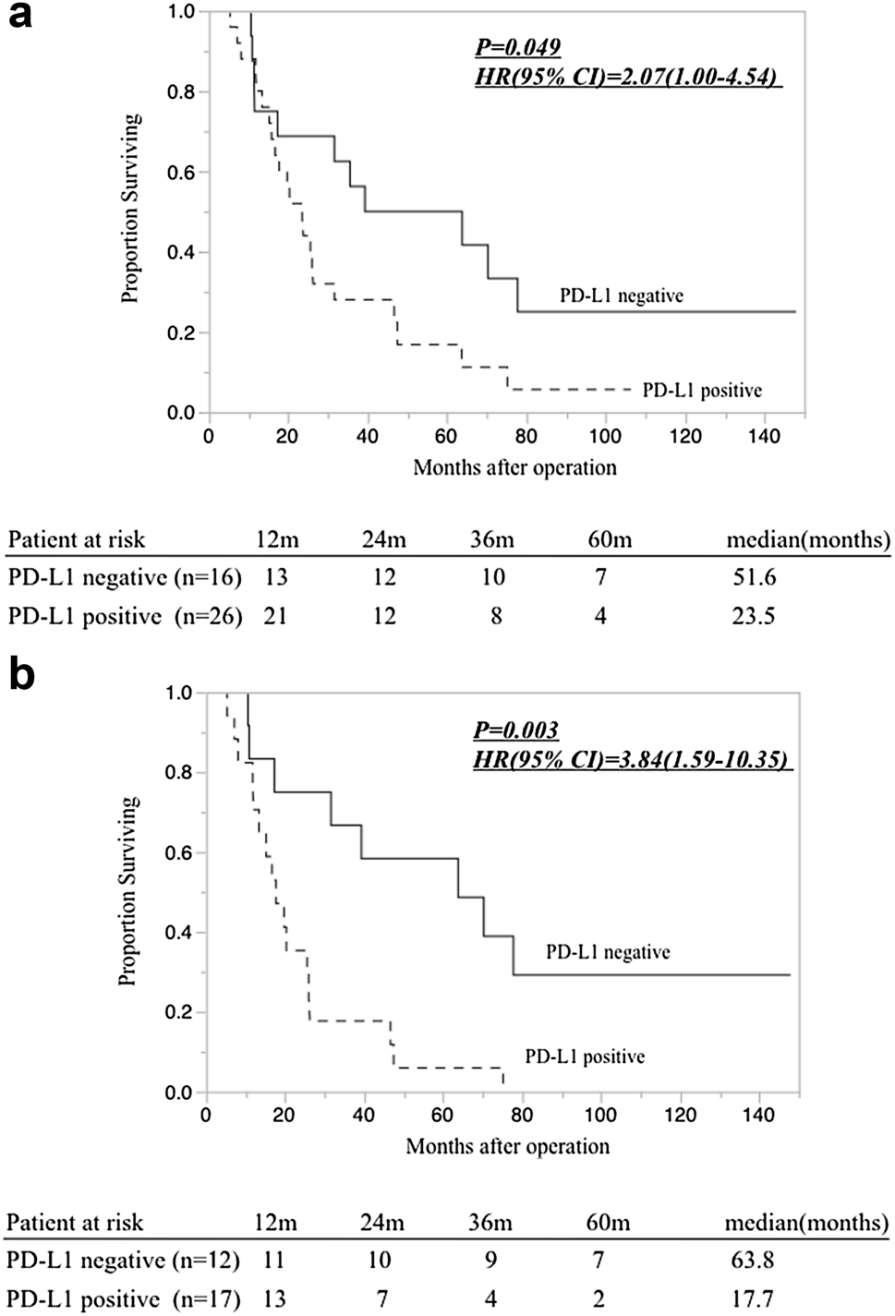
**a** Overall survival rates of 42 patients with PDAC correlated to PD-L1 expression. Overall survival in patients with PD-L1-positive disease was significantly poorer than that in patients with PD-L1-negative disease (*P* = 0.049). **b** Overall survival rate of 29 patients showing positive CD8^+^ tumor-infiltrating lymphocytes (TILs) correlated to PD-L1 expression; there was a significant difference in prognosis between patients with PD-L1-positive and PD-L1-negative disease (*P* = 0.003).* HR* Hazard ratio,* CI* confidence interval

Survival curves obtained using the Kaplan–Meier method are shown in Fig. 4. Among the 42 patients, there was a significant difference in the overall survival rate between patients with PD-L1-positive disease and those with PD-L1-negative disease based on PID staining (Fig. 4a. HR 2.07, 95% CI 1.00–4.54; *P* = 0.049). The MST was 23.5 months in the PD-L1-positive group and 51.6 months in the PDL-1-negative group. Among the 29 patients who had positive CD8^+^ TILs (>3 cells per 400× field), those in the PD-L1-positive group showed a significantly poorer overall survival rate than those in the PD-L1-negative group based on detection with the PID method (Fig. 4b. HR 3.84, 95% CI 1.59–10.35; *P* = 0.003).

The results of the univariate and multivariate analyses in the 42 patients are shown in Table 3. Univariate and multivariate analyses revealed that PD-L1 expression determined by the PID method (PID staining value >3.0) was an independent prognostic factor (HR 2.34, 95% CI 1.02–5.74; *P* = 0.045); specifically, in the 29 TIL-positive patients, PD-L1 expression determined by the PID method was an independent predictive poor prognostic factor (Table 4). Moreover, there was a stronger prognostic effect of PD-L1 expression among these 29 TIL-positive cases compared to the analysis including all 42 patients (HR 4.39, 95% CI 1.64–13.34; *P* = 0.003).

**Table 3:** Results of the univariate and multivariate analyses for overall survival in all patients (*n* = 42)

Variables^a^	Univariate analysis		Multivariate analysis	
	HR (95% CI)	*P*	HR (95% CI)	*P*
R0/1	1.81 (0.87–3.64)	0.108	1.16 (0.51–2.59)	0.710
Neo-adjuvant therapy (+/−)	1.75 (0.74–5.18)	0.220		
Adjuvant chemotherapy (+/−)	1.28 (0.64–2.61)	0.480		
Tumor marker CA19-9 > 138	1.57 (0.76–3.22)	0.216		
T stage (½, 3)	1.28 (0.58–3.21)	0.555		
N stage (0/1)	2.06 (1.01–4.44)	0.046	2.21 (0.91–5.62)	0.081
M stage (0/1)	2.25 (0.88–5.08)	0.087	1.09 (0.38–2.86)	0.863
ly (0–1/2–3)	1.23 (0.60–2.65)	0.573		
v (0–1/2–3)	1.15 (0.57–2.50)	0.697		
PD-L1 expression (+) (PID)	2.07 (1.00–4.54)	0.049	2.34 (1.02–5.74)	0.045
PD-L1 expression (+) (DAB)	1.28 (0.50–4.33)	0.633		
CD8^+^ TILs >3.0	1.31 (0.62–3.15)	0.490		

*HR* Hazard ratio, *CI* confidence interval, *PID* phosphor-integrated dots, *DAB* diaminobenzidine,* TILs* ^+^tumor infiltrating lymphocytes, *ly* lymphatic invasion, *v* blood vessel invasion

^a^See footnotes to Table 1 for explanations of staging

**Table 4:** The results of the univariate and multivariate analyses for overall survival in patients with positive CD8^+ ^tumor infiltrating lymphocytes (*n* = 29)

Variables^a^	Univariate analysis		Multivariate analysis	
	HR (95% CI)	*P*	HR (95% CI)	*P*
R0/1	1.90 (0.82–4.24)	0.131	1.55 (0.63–3.77)	0.334
Neo-adjuvant therapy (+/−)	1.42 (0.53–4.88)	0.512		
Adjuvant chemotherapy (+/−)	1.00 (0.45–2.21)	0.999		
Tumor marker CA19-9 > 138	1.14 (050–2.60)	0.751		
T stage (1–2/3)	1.08 (0.39–2.60)	0.864		
N stage (0/1)	1.64 (0.73–3.94)	0.233	1.80 (0.66–5.12)	0.250
M stage (0/1)	3.09 (0.97–8.58)	0.056	1.63 (0.47–5.05)	0.420
ly (0–1/2–3)	1.18 (0.48–2.69)	0.710		
v (0–1/2–3)	1.05 (0.43–2.43)	0.904		
PD-L1 expression (+) (PID)	3.84 (1.59–10.35)	0.003	4.39(1.64-13.34)	0.003
PD-L1 expression (+) (DAB)	1.89 (0.44–5.84)	0.349		

*ly* lymphatic invasion, *v* blood vessel invasion

^a^See footnotes to Table 1 for explanations of staging

## Discussion

We report here the results of our investigation on PD-L1 expression in patients with PDAC. The PD-L1-positive detection rate in PID staining was higher than that in DAB staining, possibly due to the fact that digital immunostaining can detect proteins at lower concentrations. The higher fluorescence intensity of the nanoparticles contributes to the higher sensitivity of PID compared to DAB. In addition, quantitative analysis was possible even in cells with strong positive expression, without saturation.

In this study, the PD-L1 positive expression rate detected using the PID method in PDAC cases was higher than that reported in previous studies (4–49%) [15–17] using the DAB method. Among the cases in our study, no case was found to be negative for PID and positive for DAB. The PD-L1 positive expression rate was 14.3% using the DAB method and 61.9% using the PID method when the PID staining threshold value was set to 3.0. This result supported the current PD-L1 protein expression data using PID, which was found to be more sensitive than DAB. However, the DAB positive staining rate was similar to the PID positive staining rate when the threshold was set to 4.0 or 5.0; in other words, cells with slightly positive expression showing three to five PID particles could not be detected by conventional methods.

Immunotherapeutic approaches, most notably immune checkpoint inhibitors epitomized by antibodies directed against T-lymphocyte regulators, cytotoxic T-lymphocyte-associated protein 4 (CTLA-4), and PD-1, have demonstrated efficacy in a variety of solid tumors, including metastatic melanoma and lung cancer, and have already received U.S. Food and Drug Administration approval. PD-1/PD-Ll pathway blockade has resulted in significant and durable clinical responses in patients with several malignancies, such as melanoma, renal cell carcinoma, lung cancer, mismatch repair-deficient colorectal cancer, and bladder cancer [18]. However, PDAC has generally been considered to be a non-immunogenic malignancy, given that tumor-infiltrating effector T lymphocytes do not represent a histopathological hallmark of this disease [19, 20]. Investigators have been actively exploring the mechanisms underlying the evasion of immune surveillance by pancreatic cancer cells, and several potential strategies have been proposed to overcome resistance to immune checkpoint inhibitors. In this study, TILs reached tumor cells in 29 of 42 surgical PDAC specimens.

In a clinical setting, patients with positive PD-L1 expression tend to show significantly unfavorable outcomes. Previous studies using DAB methods have also demonstrated that patients with PD-L1-positive PDAC showed unfavorable outcomes. Wang et al. reported a correlation between B7-H1 (PD-L1) expression and pathological grade and TNM stage [16]. Nomi et al. reported that PD-L1-positive PDAC patients had a significantly poorer prognosis than PD-L1-negative patients [17]. Their data were similar to our results in that they found was no significant correlation between tumor PD-L1 status and clinical indicators, including tumor status, nodal status, metastatic status, and pathological stage. In the current study, among the TIL-positive patients there was PD-L1 expression which showed a stronger prognostic correlation than that observed in the analysis including all patients. Univariate and multivariate analyses showed that positive PD-L1 expression (PID staining value >3.0) was indeed an independent poor prognostic factor in PDAC patients with positive CD8^+^ TILs. This result suggests that an immunosuppressive tumor microenvironment with high PD-L1 expression can interfere with the attack by TILs on the tumor cells. In these cases, there is a possibility that blockage of the PD-1/PD-L1 signaling pathway can result in the attack of TILs being dramatically more effective. Although there have been no objective responses observed in patients with PDAC who received anti-PD-L1 antibody therapy [21], our results suggest that PD-L1-positive patients with TILs may be good candidates for anti-PD-L1 antibody therapy.

## Conclusions

Phosphor-integrated dot staining provided superior results compared to those obtained by the canonical DAB staining method. We have shown for the first time that PD-L1, detected using PID staining, is a novel prognostic marker for human PDAC. Digital immunostaining is a promising tool for companion diagnostics to evaluate the therapeutic effects of molecular-targeted drugs and immunotherapy.
